# Twin Identification over Viewpoint Change: A Deep Convolutional Neural Network Surpasses Humans

**DOI:** 10.1145/3609224

**Published:** 2023-07

**Authors:** CONNOR J. PARDE, VIRGINIA E. STREHLE, VIVEKJYOTI BANERJEE, YING HU, JACQUELINE G. CAVAZOS, CARLOS D. CASTILLO, ALICE J. O’TOOLE

**Affiliations:** School of Behavioral and Brain Sciences, The University of Texas at Dallas, USA; University of Maryland Institute of Advanced Computer Studies, University of Maryland, USA; School of Behavioral and Brain Sciences, The University of Texas at Dallas, USA; School of Education, University of California Irvine, USA; Whiting School of Engineering, Johns Hopkins University, USA; School of Behavioral and Brain Sciences, The University of Texas at Dallas, USA

**Keywords:** Face recognition, deep convolutional neural network, human face recognition, human-machine comparison

## Abstract

Deep convolutional neural networks (DCNNs) have achieved human-level accuracy in face identification (Phillips et al., 2018), though it is unclear how accurately they discriminate highly-similar faces. Here, humans and a DCNN performed a challenging face-identity matching task that included identical twins. Participants (*N* = 87) viewed pairs of face images of three types: same-identity, general imposters (different identities from similar demographic groups), and twin imposters (identical twin siblings). The task was to determine whether the pairs showed the same person or different people. Identity comparisons were tested in three viewpoint-disparity conditions: frontal to frontal, frontal to 45° profile, and frontal to 90°profile. Accuracy for discriminating matched-identity pairs from twin-imposter pairs and general-imposter pairs was assessed in each viewpoint-disparity condition. Humans were more accurate for general-imposter pairs than twin-imposter pairs, and accuracy declined with increased viewpoint disparity between the images in a pair. A DCNN trained for face identification (Ranjan et al., 2018) was tested on the same image pairs presented to humans. Machine performance mirrored the pattern of human accuracy, but with performance at or above all humans in all but one condition. Human and machine similarity scores were compared across all image-pair types. This item-level analysis showed that human and machine similarity ratings correlated significantly in six of nine image-pair types [range *r* = 0.38 to *r* = 0.63], suggesting general accord between the perception of face similarity by humans and the DCNN. These findings also contribute to our understanding of DCNN performance for discriminating high-resemblance faces, demonstrate that the DCNN performs at a level at or above humans, and suggest a degree of parity between the features used by humans and the DCNN.

## 1 INTRODUCTION

**Deep convolutional neural networks (DCNNs)** now achieve human levels of accuracy on a variety of face recognition tests (for a review, see Reference [39]). For high-quality frontal images of faces, DCNNs perform at the level of professional forensic face examiners, whose identification decisions can be used as evidence in court proceedings [47]. Human–machine comparisons have been common in face recognition research for more than a decade (cf. Reference [46]). Most comparisons employ tests of face-identity matching (identity verification), whereby humans and machines decide whether two images show the “same person” or “different people.” This task is commonly performed in security, law enforcement, and forensic applications [17, 24, 30]. Because humans and machines perform many face-recognition tasks at high levels of accuracy (though see References [6, 25, 34, 38, 49], Section 5), comparison studies require face stimuli that challenge both systems.

In the present study, we compare identity verification of identical twins by humans and a DCNN trained for face identification. Identical twins provide an extreme example of the difficult conditions humans (and machines) encounter in the real world when distinguishing between highly similar faces. Human expertise for faces has long been characterized as an ability to distinguish among large numbers of highly similar faces (e.g., Reference [10]). For humans, this expertise transcends the use of local feature information to rely also on subtle differences in the configural structure of the face (cf., Reference [31]). Although this strategy is effective for most faces, it is less clear which features are most diagnostic for twin identification (cf. References [5, 35, 55, 59]; for a review, see also Section 1.1).

Because identical twins comprise roughly 1 in 250 births, establishing the unique identity of individuals in identical twin pairs is also a pressing problem for face recognition systems used in security applications (e.g., passport control). This is especially true now that these systems have been scaled up to deal with millions of individual identities. Understanding the extent to which human- and/or machine-based face recognition is reliable for highly similar faces, including twins, has both theoretical and applied value and can offer insight into the differences between the two face-identification systems.

### 1.1 Distinguishing Identical Twins

Identical twins, referred to as **monozygotic (MZ)** twins, are a sibling pair that originate from the same egg and are fertilized by the same sperm. Therefore, the pair shares 100% of their genetic make up [18], making it impossible to distinguish between a pair of identical twins based purely on DNA. Despite MZ twins sharing identical DNA, variations in appearance and/or susceptibility to diseases can emerge within the pair. These differences are due to epigenetics or to additional changes to the genetic sequence that affect how a gene is expressed [62]. Epigenetic differences become more apparent as MZ twins age due to increased exposure to different environments. For example, 3-year-old MZ twins show a more similar pattern of DNA methylation, a common epigenetic process in which a methyl (CH_3_) group is either added or removed, compared to 50-year-old MZ twins [16]. Finding variations in MZ twins does not always require extensive background knowledge of biology or genetics. There can also be phenotypic differences in the physical appearance of twins.

Techniques that detect variation in a twin pair that results as a by-product of epigenetics or physical differences between the two individuals have been used to differentiate identical twins. Iris texture, for example, does not depend on genetics. It has been found that iris texture is stable at as early as 8 months gestation, remains so across the lifespan [27, 58], and can be differentiated by humans with more than 81% accuracy with only 3 seconds of viewing time [23]. Identical twins also have different fingerprints. By 7 months of age, the patterns on the fingers are fully developed and serve as a reliable measure of identity [24, 58]. Although iris scans and fingerprints can provide reliable biometrics for identical twins, neither is easily accessible for identity verification in security scenarios (e.g., passport control).

Face recognition of MZ twin pairs relies on observable phenotypic variations within the twin pair. As noted, these phenotypic variations become more apparent as diverging epigenetic processes ensue. Changes in the appearance of the face can be due to natural effects of aging or by certain lifestyle-related behaviors. The face naturally changes across the lifespan [15]. For example, the skeletal structure of the face changes over time, altering an individual’s face shape across the lifespan. Changes in the skin, like the deflation of subcutaneous fat and changes in musculature, also affect the appearance of the face. In addition, certain behaviors, such as smoking, can lead to more rapid changes in the appearance of the skin due to epidermal and dermal thinning, making the skin appear more droopy [15, 19, 52]. These features of facial aging combine to make twin faces more easily discriminable after the age of 40 [45, 52].

Recently, computational approaches have been considered for extracting the face features that are diagnostic for discriminating identical twins. To locate “critical features” in pairs of images that depict the faces of identical twins, a Modified Scale Invariant Feature Transform [29] was applied to determine mismatched facial key points between the image pairs [35]. The points were then overlaid on five facial landmarks: eyes, eyebrows, nose, mouth, and face curve. The “face curve” (i.e., the outline of the lower face) contained the largest number of mis-matched points and was, therefore, considered the most diagnostic face region. Human ratings of the image pairs converged with the algorithm with “face curve” ranked as the most diagnostic feature in approximately 35% of twins’ face images [35]. However, this finding is at odds with an earlier study in which facial markings (e.g., scars, moles, freckles) were rated as the most useful features (Reference [5], Section 1.2). Moreover, using a twin discrimination algorithm based solely on facial markings showed that these markings are correlated across twin pairs [55], thereby making their value less clear.

### 1.2 Computer-based Face Recognition of Monozygotic Twins

In this section, we provide a brief history of studies examining the application of automatic face-recognition algorithms to the problem of differentiating identical twins. For a comprehensive review of this literature, see Reference [59].

#### 1.2.1 Pre-DCNN Algorithms.

Experiments testing the performance of commercial face-recognition algorithms between 2011 and 2014 concluded that face-recognition technology could not distinguish identical twins [7]. Computer-based face-identification systems at that time typically used either **principal component analysis (PCA)** or hand-selected features to process face images [36, 61] and employed log-likelihood functions to reduce the error rate [53].

The studies reviewed in this section rely almost exclusively on face images from the Notre Dame Twins dataset (ND-TWINS-2009–2010) [48]. This database contains images of identical twins, fraternal twins, and sibling pairs taken at various poses and under different illumination conditions. Although another twin database was available at that time [58], ND-TWINS-2009–2010 includes racial diversity and contains far more identities. Notably, for some twin pairs, images are available from photographs taken in 2009 and 2010, thereby supporting timelapsed recognition tests. The availability and quality of this dataset has spurred multiple studies of twin face recognition.

In one of the first papers to compare human and machine performance on twin identification [5], human accuracy and strategy were studied with the goal of understanding (and potentially implementing in future work) successful methods used by human participants when distinguishing identical twins. Participants completed an identity-verification task in which they viewed pairs of identical-twin siblings (different-identity trials) and an equal number of same-identity image pairs. All pairs of images were taken under comparable illumination conditions. Participants rated the likelihood that the pair of images showed either the same person or identical-twin siblings, using a 5-point scale ranging from (1) “Sure they are the same person” to (5) “Sure they are identical twins.” Humans performed substantially better when they were given more time to make a decision (average accuracy = 92.88%) versus when they examined image pairs for only 2 seconds (average accuracy = 78.82%). This time dependency does not apply to non-twin faces, which are identified as accurately in 2 seconds as with unlimited time [41]. Additionally, people were more accurate for twin pairs with facial markings. Among the computer algorithms tested, only one commercial algorithm (Cognitec) performed at a level that approached, but was below, human performance.

In subsequent work with pre-DCNN systems, the focus of algorithm tests was on the extent to which image variation (e.g., variation in illumination, pose, and expression) affected face identification in twins. Three commercially available face-verification systems (Cognitec 8.3.2.0, VeriLook 4.0, and PittPatt 4.2.1) and a face-verification system based on **Local Region Principal Component Analysis (LRPCA)** were tested on front-facing images of identical twins that varied in expression and illumination [48]. Of the commercial face-verification systems, two performed well on the controlled-illumination and neutral-expression test (Cognitec and VeriLook). This was likely due to the sensitivity of these systems to high-resolution texture features in the face. PittPatt, which was optimized for small faces, performed poorly, as did the baseline LRPCA. However, the two face-identification systems that performed well for face images taken under the same conditions showed high false-alarm rates when image conditions varied [48]. In related work [45], the performance of seven commercial algorithms was examined across variations in illumination, expression, gender, and age for both the same-day and cross-year images [42]. Three of the algorithms tested were among the top submissions to the Multiple Biometric Evaluation 2010 Still Face Track. Performance varied widely among the algorithms, though all algorithms performed less accurately when distinguishing identical twins versus non-twins.

In general, the conclusion of this work is that twin recognition was achievable with pre-DCNN algorithms when the comparison images were well matched. This is consistent with the more general face recognition literature at that time, which consistently showed for algorithms that the closer the match in terms of image (e.g., similarity in pose, illumination, and expression) and appearance, the more accurate the performance of an algorithm. This also applies to human face recognition performance, which differs as a function of the similarity of image and appearance conditions in image pairs (e.g., References [8, 40]). This early literature also showed that human participants identified identical twins far better than algorithms at that time.

#### 1.2.2 Deep-learning Approaches.

DCNNs [26] have been remarkably successful in advancing the state of the art in automatic face recognition (e.g., References [12, 13, 51, 54, 57, 60]). A strong advantage of these networks is their ability to generalize across image and appearance variation. There have been only a few attempts to apply DCNN-related technologies to the problem of identifying twins [2, 3, 33, 56]. These studies are difficult to compare to each other and to the previous literature, because they typically used twin datasets that differ from those used in the pre-DCNN era and because these datasets are not entirely accessible. Moreover, they use DCNNs with diverse architectures and goals. In what follows, we briefly summarize these studies.

In one study [2], a combination of local feature extraction algorithms based on PCA, **Histogram of Oriented Gradients (HOG)**, and **local Binary Patterns (LBP)** performed more accurately than an object-trained CNN on the ND-TWINS-2009–2010. In a subsequent study [33], the goal was to create a baseline facial similarity measure between identical twins and to use this baseline to measure the impact of “look-alike” identities with no familial relationship. To that end, a larger face dataset was created by combining the twin dataset presented in Reference [42] and CelebA [28]. A Siamese DCNN with a FaceNet architecture was trained to minimize the L2 distance between similar samples and to maximize L2 distance between dissimilar samples in the feature space. The output similarity score was the L2 distance between two samples in the feature space. The mean similarity score across all twin pairs served as the baseline facial similarity between identical twins. Next, the authors used the DCNN to process a large-scale non-twin dataset. They found that 6,153 of the 15,455 identities (39.8%) had one or more potential look-alikes, as defined by the aforementioned threshold. Furthermore, 288 identities (1.475%) had one or more potential look-alike above the fourth quartile threshold. The authors suggest using this similarity score to identify potential look-alikes in large-scale datasets to extract difficult cases for a facial recognition system or for intelligent morph-pair generation.

In a second study [56], a DCNN was trained first on a large dataset and was subsequently optimized to distinguish between identical twins. The model was tested on the **Twin Days Festival Collection (TDFC)**, with high-quality frontal face images of twins. The performance of the network was promising, with error rates of between 9.4% and 13.8%. This study showed the plausibility of applying deep networks optimized for twin discrimination to the problem of identifying twins. One limit of the study, however, is that the dataset tested (TDFC) is not available to the public for replication and viewing. Thus, it is difficult to interpret the results of the experiments in terms of the challenge level of the stimuli.

More recently, the problem of distinguishing the faces of identical twins was examined in the **Face Recognition Vendor Test (FRVT)** conducted by the **National Institute of Standards and Technology (NIST)** [20]. That study addressed the question of whether identical twins could impersonate each other (for example, at a border crossing). The FRVT study tested multiple algorithms from corporate research, development laboratories, and universities, using identical twin faces from two sources: (a) a dataset from West Virginia University taken at the Twin Days Festival in Ohio (2010–2018) and (b) a U.S. Government database in operational travel and immigration. Accuracy at discriminating the twin pairs was measured in three steps as follows: (1) algorithmgenerated similarity scores were computed for a set of mugshots; (2) using these similarity scores, the threshold that produced a false alarm rate of 1 in 10,000 was found; and (3) this threshold was applied to determine the false alarm rate for the identical twin pairs (i.e., similarity between the face images of two twins). The results indicated that none of the algorithms submitted to the FRVT could detect an identical-twin imposter at a threshold set to produce 1 in 10,000 false alarms.

Although the FRVT provides a valuable look at the state of the art for the ability of face-identification applications to detect twins impersonating each other, the conclusions that can be gleaned from the results of the study are limited for several reasons. First, because of the focus on impersonation, the FRVT’s accuracy measure utilized only different-identity twin pairs. Second, the systems evaluated are largely proprietary, and so the underlying procedures and technical components of the algorithms are unknown. Third, the datasets used to test the algorithms are not available to the research community. This precludes replication of NIST’s results and bench-marking other algorithms with the same images.

In the present study, we measured face verification in a more general way, using both same-identity (two different images of the same twin) and different-identity image pairs (images of identical twins or of two demographically matched un-related identities). This supports the computation of an **area under the receiver operating characteristic curve (AUC)**, which considers performance across all thresholds. We also evaluated an algorithm published in a peer-reviewed paper [51] using a dataset that is freely available to researchers.

### 1.3 Experimental Goals

The goal of the current project was to compare the identification accuracy of humans and a high-performing DCNN on a test that included identical twins and to test performance across changes in viewpoint. This provides a human benchmark test on exactly same face stimuli and viewpoint conditions used in the algorithm test. As noted, a strong advantage of face identification DCNNs over previous generations of algorithms is that they show an ability to generalize identification over changes in viewpoint and illumination (cf. for a review, see Reference [39]). Accordingly, in Experiment 1, we designed a face-identity matching experiment in which humans judged the likelihood that pairs of frontal-to-frontal, frontal-to-three-quarter (45°) profile, and frontal-to-full (90°) profile images showed the same person or different people. Image pairs showed either two different images of the same person, images of identical twins, or images of different people of the same gender, race, and approximate age. Because most face recognition algorithms operate on an internal face crop (including the one tested here), face images for the human experiments were cropped to limit the available visual information to only the internal face. This assured parity between the information available to the humans and machine.

In Experiment 2, we tested a DCNN on the same task. We chose a network [51] that performed at a level of accuracy comparable to professional forensic face examiners and super-recognizers [47]. We also tested whether there was a relationship between the perception of highly similar images by humans and the DCNN by correlating human and machine ratings of similarity. Note that exactly the same internal face crop images were (a) shown to the human participants in Experiment 1 and (b) processed by the face-identification DCNN tested in Experiment 2.

## 2 EXPERIMENT 1: HUMAN RECOGNITION OF IDENTICAL TWINS

In Experiment 1, we measured twin identification performance in human participants using the ND-TWINS-2009–2010 dataset.

### 2.1 Methods

#### 2.1.1 Participants.

A total of 87 student participants were recruited from the **University of Texas at Dallas (UTD)**.^1^ Participants were compensated with class credit in exchange for their time. For each viewpoint condition (frontal-to-frontal, frontal-to-45°, and frontal-to-90°), there were 29 participants. Participants were required to be at least 18 years old and have normal or corrected-to-normal vision. Eligibility was determined through self-report using a Qualtrics survey. All experimental procedures were approved by the UTD Institutional Review Board.^2^

#### 2.1.2 Experimental Design.

Face-identity matching (identity verification) from pairs of images was tested as a function of the type of stimulus. Image pairs showed either the same identity (same-identity pairs) or different identities. In the latter case, the different-identity pairs were either twin-imposter pairs or general-imposter pairs. *Same-identity pairs* consisted of two different images of the same identity. *Twin-imposter pairs* consisted of identical twin siblings. *General-imposter* pairs consisted of two images of different people who were not related to one another. Each of these pair types was tested in each of the three viewpoint conditions.

Identity-matching accuracy was measured by computing the AUC for two conditions: (a) same-identity pairs versus twin-imposter pairs and (b) same-identity pairs versus general-imposter pairs.

#### 2.1.3 Stimuli.

Images were selected from the ND-TWINS-2009–2010 database [48]. The database contains 24,050 images of 435 different identities. Identities in the database are of sibling sets of one of the following types: identical twins, fraternal twins, non-twin siblings, and identical triplets. Identities in the database were photographed in both indoor and outdoor illumination conditions. Multiple viewpoints of each face were available for most identities, including −90°, −45°, 0°, 45°, and 90° views.

The stimulus set we used contained 100 face-image pairs of Caucasian identities and 20 face-image pairs of African-American identities. Although the dataset included both Caucasian and African-American faces, there were too few African-American identities to create balanced experimental conditions. In each condition, we tested an equal number of matched-illumination pairs (indoor–indoor) and non-matched illumination pairs (indoor–outdoor). The resulting sample included 240 images of 200 identities, from which we formed 40 same-identity pairs, 40 twin-imposter pairs, and 40 general-imposter pairs (120 trials). Across all trials, identities appeared only once to prevent participants from becoming familiar with any given identity. All faces were cropped to include only the inner-face region, with minimal hair visible.

Image pairs in the general-imposter condition were composed of two individuals of the same gender and race, with an age difference of no more than 8 years. To maximize the number of identity pairs available for inclusion, general-imposter pairs were selected from the full range of identities in the dataset regardless of an identity’s sibling type.

**Fig. 1.**
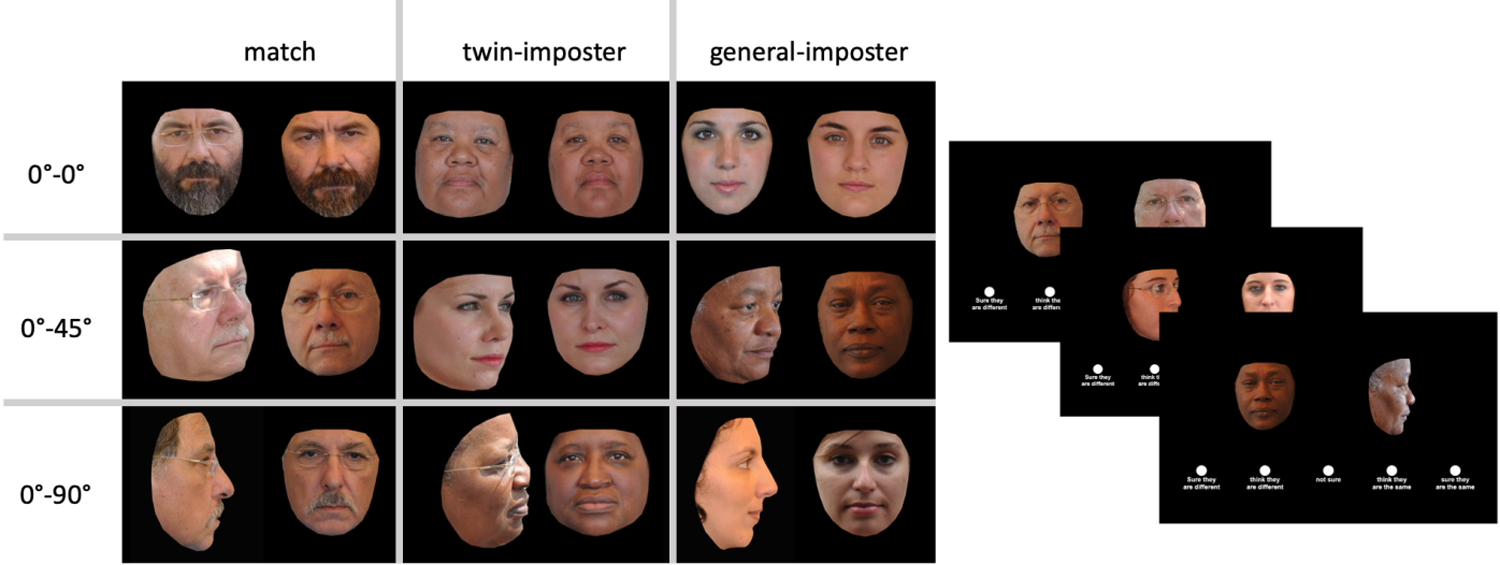
(Left) Examples of face-image pairs viewed during Experiment 1. The first column shows examples of same-identity pairs (match pairs), the second column shows examples of twin-imposter pairs (identical twins), and the third column shows examples of general-imposter pairs (unrelated individuals). The viewpoint-disparity conditions are shown along the rows, defined as frontal to frontal (top), frontal to 45° (middle), and frontal to 90° (bottom). (Right) The frame cascade depicts the experimental trials as seen by human participants. Each frame in the cascade depicts a different viewpoint-disparity condition.

For the frontal-to-45° and frontal-to-90° viewpoint disparity conditions, we began with the image pair used in the frontal-to-frontal condition. For each pair, we retained one (randomly selected) frontal image and replaced the second image with a 45° or 90° profile that matched the image parameters of the image being replaced. Example image pairs appear in Figure 1.

#### 2.1.4 Procedure.

Participants first completed a screening survey to determine eligibility. The survey confirmed that participants were at least 18 years old and had normal or corrected-to-normal vision. If participants satisfied the criteria, then they were directed to an online informed consent form. Participants completed the informed consent form and then were given an access code to schedule a study time slot. During their scheduled time slot, the participant met with a research assistant over Microsoft Teams, using a link specific to the participant.

The researcher briefly described the task by explaining to the participant that they would view a series of face pairs and rate their certainty about whether each image pair showed the same person or two different people.^3^ Participants were told that there may be identical twins present in the experiment.

On each trial, a pair of face images appeared side by side on the screen. Participants were asked to determine whether the image pairs showed the same person or two different people using a 5-point scale. The response options included: (1) “Sure they are different,” (2) “Think they are different,” (3) “Not Sure,” (4) “Think they are the same,” and (5) “Sure they are the same.” Responses were reported by using a mouse to select the rating. The images and scale remained on the screen until the participant selected a response. The experiment was programmed in PsychoPy. The presentation order of the trials was randomized for each participant.^4^

### 2.2 Human Results

#### 2.2.1 Accuracy.

Identity-matching accuracy was measured by computing the AUC. For each participant in each viewpoint condition, an AUC was computed for (a) image pairs viewed in the general-imposter condition and (b) image pairs viewed in the twin-imposter condition. For both the general-imposter condition and the twin-imposter conditions, correct identity verification responses were generated from same-identity image pairs. In the general-imposter condition, false alarms were generated from image pairs that showed two distinct, unrelated identities. In the twin-imposter condition, false alarms were generated from image pairs that showed identical twins. Because the distribution of correct verification responses was the same for both the general-imposter condition and the twin-imposter condition, the difference in the AUC between these conditions is due to identity-matching ability on the different-identity (twin-imposter and general-imposter) pairs.

**Fig. 2.**
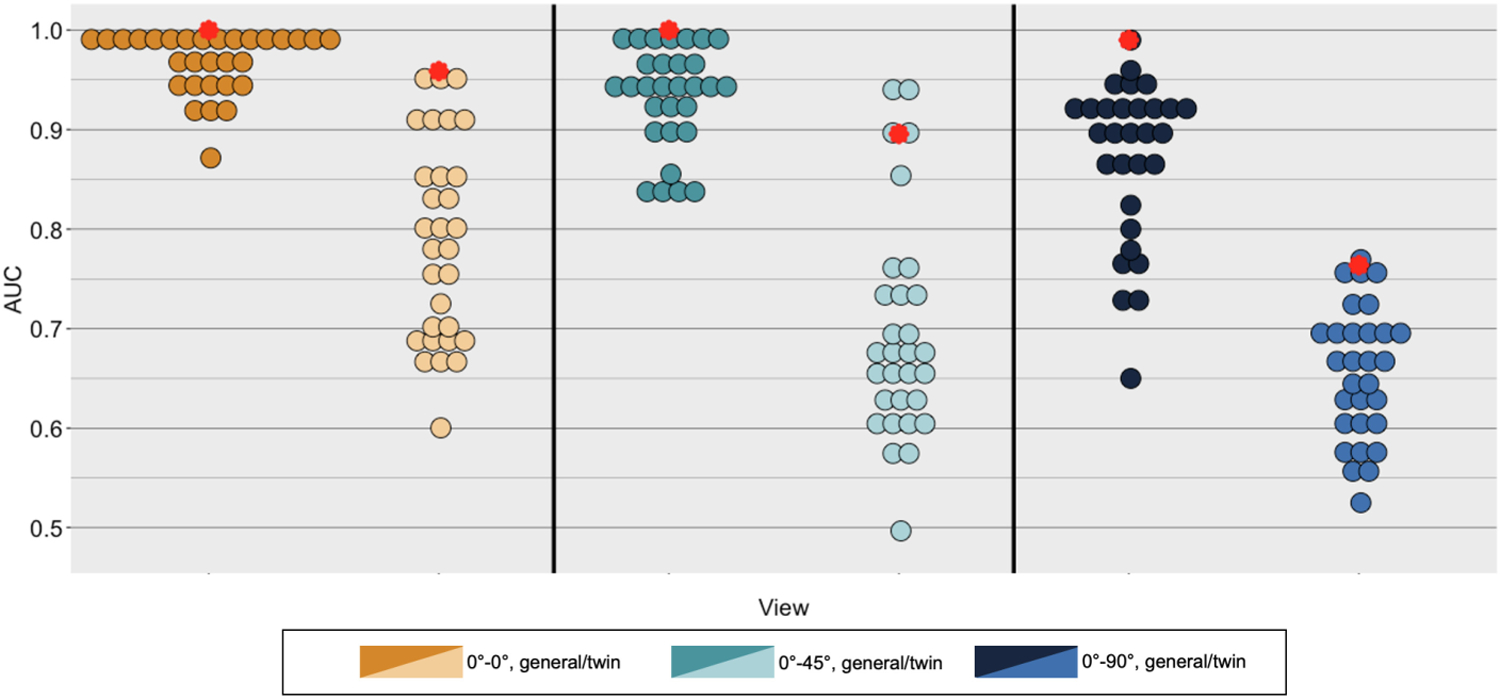
Identification accuracy for the DCNN and human participants. Both exhibited lower accuracy for twin identification, and both show a decline in performance as viewpoint disparity increases. The DCNN outperformed or performed at a level comparable to the highest-performing humans in all conditions. (Red circles represent DCNN performance. Other circles represent individual human-participant accuracy.)

Figure 2 shows a violin plot of human accuracy for the general- and twin-imposter conditions across viewpoint. The average AUC scores across humans by condition from left to right are as follows: general imposter, frontal-to-full (0.969); twin imposter, frontal-to-frontal (0.874); general imposter, frontal-to-45° profile (0.933); twin imposter, frontal-to-45° profile (0.691); general imposter, frontal-to-90° profile (0.869); and twin imposter, frontal-to-90° profile (0.652). More formally, the AUC data were submitted to a two-factor analysis of variance with the twin condition (within-subjects) and viewpoint (between-subjects) as independent variables. As expected, performance was more accurate for the general-imposter pairs than the twin-imposter pairs, *F* (1, 84) = 649.84,*p* ≈ 0.00, $\eta_{G}^{2}=.28$. Performance also differed as a function of viewpoint disparity *F* (2, 84) = 22.80,*p* ≈ 0.00, $\eta_{G}^{2}=.67$. There was a significant interaction between viewpoint and twin condition, *F* (1, 84) = 3.71, *p* = 0.003, $\eta_{G}^{2}=.02$. The pattern of means suggests that performance declined more rapidly as a function of viewpoint change for the twin imposters than for the general imposters.

In summary, in all conditions, identity-matching was substantially more accurate for the general-imposter condition than for the twin-imposter condition. Accuracy decreased as a function of the viewpoint disparity between the images, with this decrease more pronounced for the twin-imposter condition than the general-imposter condition.

## 3 DCNN RECOGNITION OF IDENTICAL TWINS

### 3.1 Methods

#### 3.1.1 Network Architecture.

For the algorithm test, we used a DCNN based on the ResNet-101 architecture [21, 50]. The network was trained on the Universe dataset, which is a web-scraped, “in-the-wild” dataset containing 5,714,444 images of 58,020 unique identities [4]. Images in this training dataset are sampled to include large variation in image attributes, including pose, illumination, resolution, and age [4]. The demographic composition of the Universe dataset used to train the network is not known. The network contains 101 layers and employs skip-connections to maintain the amplitude of the error signal during training. Crystal loss is applied to ensure that *L*_2_-norm is held constant during learning, and the alpha parameter is set to 50. In addition, as a pre-processing step for network training, face images were cropped to include the internal face and aligned to the size of 128 × 128 prior to being input to the network. This procedure was applied in the same way for all image poses. When the fully trained network is complete, the output of the penultimate fully connected layer is used to generate identity descriptors for each image processed through the network. The resulting network is a high-performing face-identification system that maintains accuracy across substantial changes in viewpoint, illumination, and expression (cf. Reference [32]).

#### 3.1.2 Similarity Scores.

To generate data that can be compared to humans, each face image presented to the human participants was processed through the DCNN to produce a descriptor for the image. Note that these images were cropped around the internal face and presented to the network in the same form as they were presented to the participants. To assess the similarity of the DCNN representation of the image pairs, we computed the cosine angle (i.e., normalized dot product) between the corresponding identity descriptors.

### 3.2 Results: DCNN Identification Accuracy

DCNN-based similarity scores were generated for each of the image pairs viewed during the human data collection experiment. Identification accuracy for the DCNN was measured by computing the AUC for the similarity scores assigned to same-identity image pairs and different-identity images pairs. Correct responses were generated from image pairs showing the same identity, and false alarms were generated from image pairs showing different identities. DCNN performance is shown in Figure 2 as a red circle, overlaid on the individual human performance data.

For the general-imposter condition, the DCNN obtained perfect identity-matching performance (AUC = 1.0). For the twin-imposter condition, DCNN identity-matching performance remained high (AUC = 0.96).

## 4 HUMAN AND MACHINE PERFORMANCE COMPARISON

**Fig. 3.**
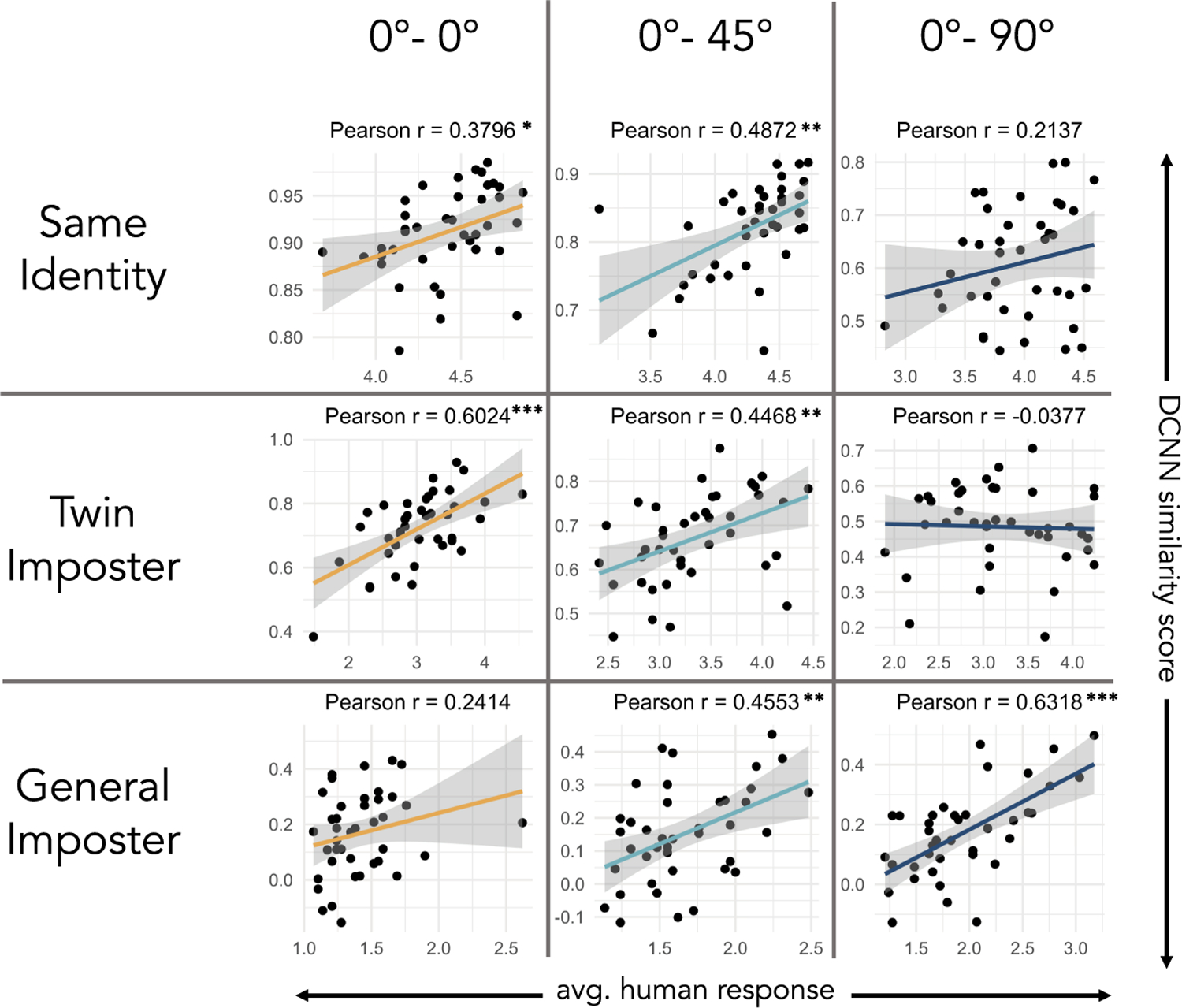
Plots depict correlations between human match ratings and the DCNN similarity score for each type of image pair. In six of nine conditions, humans and DCNN “similarity ratings” correlated significantly. The gray regions within each plot indicate the 95% confidence intervals.

To examine the relationship between human and DCNN ratings, Pearson product moment correlations were computed between average human ratings and DCNN similarity scores for all image pairs in the three viewpoint conditions. Specifically, for each item (image pair), we computed the average rating given by participants and correlated these average ratings with the DCNN similarity scores. Figure 3 shows the scatter plots comparing average human response ratings on the *x* axis and DCNN similarity scores on the *y* axis, with each point representing an image pair. Of the nine correlations, six were significant [range *r* = 0.38 to *r* = 0.63]. To examine the stability of these correlations across participants, we generated 50 “average” response-rating scores for each item by sampling half of the participants and then averaging their responses for each item. This was done for the match, general-imposter, and twin-imposter conditions. Next we generated 95% confidence intervals for the correlations in each condition. The means and confidence intervals are as follows: 0°–0° : same identity = 0.346 [*CI*, 0.316, 0.375], general imposter = 0.470 [*CI*, 0.452, 0.487]; twin imposter = 0.191 [*CI*, 0.167, 0.214]; 0–45°: same identity = 0.584 [*CI*, 0.570, 0.597]; general imposter = 0.434 [*CI*, 0.418, 0.449]; twin imposter = 0.040 [*CI*, −0.020, −.055]; 0°–90°: same identity = 0.225 [*CI*, 0.207, 0.242]; general imposter = 0.443 [*CI*, 0.427, 0.458]; = 0.616 [*CI*, 0.602, 0.629]. These confidence intervals are indicated by the gray regions shown in Figure 3.

The majority-significant correlations suggest that there is a relationship between the perceived similarity ratings from the human participants and the similarity score generated by the DCNN. Combined, the results suggest a moderately strong relationship between the model’s and humans’ assessment of facial similarity. As noted previously, our goal in testing humans was to provide a benchmark for the DCNN that was based on the same set of face images and viewpoint conditions. To the best of our knowledge, this is the first such benchmark for identical twin faces. The results shows moderate to high levels of accord between the face similarity ratings produced by humans and the DCNN across the majority of conditions. Although the underlying mechanisms and features of similarity judgments are not known for either the model or humans, given the difficulty of the task and the limited availability of facial cues that differentiate twins, the accord is consistent with the conclusion that the DCNN and humans perceive the same twin faces as (dis)similar.

## 5 GENERAL DISCUSSION

Accurately distinguishing between identical twins requires the use of epigenetic biometric features that remain relatively stable across the lifespan. Although fingerprints and iris texture have been considered the most reliable biometrics for distinguishing between identical twins [23, 24, 57, 58], facial identification can also be used to distinguish identical twins and has the advantage of being less invasive. Until the advent of DCNN algorithms, however, the best face identification algorithms performed well only when the images of twins were highly controlled and matched for imaging conditions (e.g., both frontal pose, similar illumination conditions) [5, 42, 45, 48]. Even in this controlled case, pre-DCNN algorithms perform poorly relative to humans on the task of discriminating identical twins [5].

In the present study, we tested human and DCNN performance on a face-identification task that included identical twins and required identification across large changes in viewpoint. Identical twins represent a special class of face stimuli that exhibit high similarity between different identities. The results of this study highlight the impressive progress of DCNNs over previous face-identification algorithms. We found that DCNN accuracy exceeded the accuracy of nearly all human participants tested in all conditions. Although previous findings show that DCNNs perform at or above human levels of accuracy on a variety of face-identification tasks [39], the present work extends the human–machine comparison results to the highly challenging task of twin identification over viewpoint change.

It is worth noting that the recent study by NIST [20] addressed the applied question of whether twins can impersonate one another. Their results suggested that DCNNs perform poorly with identical twins. By contrast, we tested identification in a more standard and comprehensive way by examining the AUC measure for same-identity pairs versus both general-imposter pairs and twin-imposter pairs. Our results showed that the DCNN can discriminate same-identity face pairs from different-identity twin-imposter pairs—even over large changes in viewpoint. The NIST results are not inconsistent with the present results but instead highlight the importance of considering claims about the performance of DCNNs in the context of the problem(s) addressed. Returning to the behavioral results, the inferior, but still strong, performance of humans was unexpected given the fact that these faces were unfamiliar to participants. Although humans have been considered face recognition “experts” [9, 14], it has become clear in recent years that this expertise may not generalize to “unfamiliar faces” [25, 34, 49]. For example, in identity-matching tasks such as the one we conducted here, people are far more accurate when they are familiar with the person/people in one or both of the images. For DCNNs, the face identity matching process treats all faces as unfamiliar [39]. Specifically, DCNNs are pre-trained with very large numbers of faces, but are tested with faces not used during training (cf. Reference [39]). Although DCNNs have been used to simulate “face familiarity” (e.g., References [6, 37]), there are a variety of different methods available for implementing familiarity, which have not been tested or compared systematically [39]. More work is needed to develop these methods and to link the types of mechanisms employed to human face familiarity. Notwithstanding, it is likely that familiarization with the people in the images could be used to further improve both human and machine twin identification.

A related point involves whether performance might differ depending on whether participants believe that they are looking at a pair of identical twins or whether they assume that the image pair does not potentially show twins. It is plausible that participants might have applied different strategies in these two cases. In our experiments, we took an approach whereby participants were alerted to the possibility of twins but were not led to believe that all or even most of the “different-identity” image pairs would be twins. Indeed, the general-imposter condition should have assured participants that many different-identity trials did not show identical twins. An explicit test of instructions as a variable would be needed to determine whether twin-specific strategies might affect performance for humans. Along these lines, in our experiment, we cropped the images to present only internal facial features, so that there would be parity between the information available to the network and the information available to humans. In practical terms, however, people can rely on information about external features (e.g., hair style and texture) to discriminate between identical twins in natural viewing conditions. Future work should consider these features as a source of identity-diagnostic information for twin identification.

One goal of this work was to gain insight into the relationship between face identification in humans and machines. The question of whether DCNNs perform in ways similar to humans can be considered at the level of the (a) experimental conditions, (b) individual participants, and (c) stimulus items. Starting with the experimental conditions, both the human and machine results showed the expected pattern of decreased accuracy for the twin-imposter pairs relative to the general-imposter pairs. Moreover, both also showed the expected accuracy decrease with increasing viewpoint disparity between the images in the comparison pair.

From the perspective of the individual participants, a particularly notable aspect of human performance was the wide range of accuracy across individuals. In all conditions, some participants performed at the level of the machine, others moderately below that level, and still others *substantially* below the level of the machine. This is an important finding in the context of past work on face identification variability in law enforcement and security scenarios (cf. References [11, 47, 47, 63]). DCNN performance was consistently at the top of the human performance distributions and is not subject to variability. Therefore, we might consider the “machine” to be similar to a “normal” (untrained) person who is very good at the task of face recognition. However, the best human face recognizers are forensically trained face examiners and reviewers [47, 63] and super-recognizers [38, 47]. These groups would likely be more accurate than the university students we tested. It would be of interest to measure the performance of these special populations on the test administered here.

The strongest case for establishing a degree of parity between the face “features” used by humans and machines to discriminate identical twins can be made at the level of the individual stimulus items. We found significant item-based correlations between the average human response (1:sure same to 5:sure different) and the similarity of DCNN-generated image representations in six of the nine experimental conditions. It is not possible to determine why the human–machine judgments correlated more in some conditions than in others. Moreover, the conditions that showed little-to-no correlation do not easily lend themselves to speculation. We note only that two of the three conditions in which human and machine responses were unrelated involve the largest viewpoint disparity (match and twin imposter in the 0°–90° viewpoint condition). That said, the highest human–machine correlation was obtained for the largest viewpoint disparity in the general-imposter condition. It is possible that the difficulty of these conditions pushed participants into idiosyncratic strategies that may have added noise to the responses. Notwithstanding, the significant correlations suggest that input from DCNN-based face identification systems can be used to predict the perceived similarity of an image pair as seen by human participants.

It remains a challenge going forward to come to a better understanding of the nature of the information captured in DCNN-generated representations of faces. Previous methods aimed at probing the critical features for face identification are not useful here, because they can be applied only to two-dimensional images from a single viewpoint [1, 33]. Instead, the present study used item-based correlation to compare the ability of humans and the DCNN to operate across changes in viewpoint. These correlations point to general accord in the assessment of face similarity by humans and the machine—even across viewpoint change.

Some progress has been made on understanding DCNN-generated face codes that operate across viewpoint. Specifically, computational analyses show that these representations retain detailed information both about the face identity and about the actual input image processed by the network [22, 43]. Moreover, it is now known that the representation of image viewpoint is distributed across the units in the network output, whereas identity information is strongly represented both in individual units and across units [44]. A recent review provide an overview of what is known and what still remains to be known [39].

In summary, the present study tested human and machine performance on a face identification task involving identical twins, an extremely difficult task for humans and machines alike due to the high similarity of different-identity image pairs. The DCNN surpassed or performed at the level of the best humans across all experimental conditions, indicating that DCNNs now outperform the average human on a once human-dominated task. The results demonstrate that DCNNs are becoming highly accurate in more challenging face-identification contexts, more so than humans, suggesting that difficult image-matching tasks (e.g., forensic applications) could benefit from joint human and DCNN decision making.

## CCS Concepts:

• **Computing methodologies** → *Neural networks*; **Image representations**; • **Networks** → *Network performance analysis;*

## Footnotes

1 Although we intended to collect participant demographic data, a human data collection error resulted in incomplete demographic information about participants.

2 Note that the frontal condition was tested first, due to our initial assumption that the DCNN would not perform well enough to attempt an identification test on the 45° and 90° profile images. Thus, the 45° and 90° profile test for humans was carried out after the frontal test was complete, and so human subjects were not assigned randomly to viewpoint groups.

3 Note that the viewpoint conditions (frontal-to-frontal and frontal-to-profile) were tested at different times, and there are some variations in the procedure. For the frontal to frontal condition, the researcher shared their computer screen with the participant over Microsoft Teams. Mouse control was transferred to the participant while they completed the experiment independently through the research assistant’s computer. For the frontal-to-profile conditions, participants completed the same experiment format but did so independently using Pavlovia.

4 For the frontal-to-frontal condition, participants completed an exit survey that confirmed their demographics such as age and ethnicity. This survey was not employed for the frontal-to-profile conditions.
